# Understanding the role of distance, direction and cue salience in an associative model of landmark learning

**DOI:** 10.1038/s41598-019-38525-7

**Published:** 2019-02-14

**Authors:** Sean Commins, Dirk Fey

**Affiliations:** 1 Department of Psychology, Maynooth University, Maynooth, Co. Kildare Ireland; 2 Systems Biology Ireland Conway Institute, University College Dublin, Dublin, Ireland

## Abstract

Navigation and spatial memory relies on the ability to use and recall environmental landmarks relative to important locations. Such learning is thought to result from the strengthening of associations between the goal location and environmental cues. Factors that contribute to the strength of this association include cue stability, saliency and cue location. Here we combine an autoregressive random walk model, that describes goal-directed swimming behaviour, with an associative learning model to provide an integrated model of landmark learning, using the water maze task. The model allows for the contribution of each cue, the salience and the vector information provided (both distance and directional) to be separately analysed. The model suggests that direction and distance information are independent components and can influence searching patterns. Importantly, the model can also be used to simulate various experimental scenarios to understand what has been learnt in relation to the cues, thereby offering new insights into how animals navigate.

## Introduction

Learning to navigate accurately is essential for many animals, but how this is achieved is currently a matter of debate. Cognitive map theory^1^ suggests that an animal, following exploration of an environment, develops a map-like representation using environmental cues/landmarks. From this, the animal can create novel routes and shortcuts to the goal. A more recent account suggests that successful navigation may be attributed to a simpler associative mechanism^2^, whereby environmental cue(s) become associated with the goal^3,4^. With this model, animals need only to associate certain elements of the environment as required. There is no need to formulate a map of the entire environment. As such, some cues may be weighted as more important than others^5^; all elements of the environment are not treated equally. One popular tool used to study spatial navigation and memory is the Morris water maze^6^. This task requires animals to escape from a pool of water by finding a platform, hidden just below the surface. As animals can not see the platform directly, they must use the cues surrounding the maze to find the goal^7^. This learning seems to be well accounted for by an associative mechanism. For example, overshadowing and blocking, two important components of associative learning, have been demonstrated in water maze learning^4,5,8^. In addition, we have demonstrated that animals use individual cues as a visual guide towards the hidden platform^9^; all cues are not equally important^2,10^. Although animals seem to use an associative mechanism in spatial learning, there is no clear indication of how this is achieved. For example, if some cues are considered more important, how are these ‘chosen’? Cue salience may play an important role^11^. Specific features, including cue size, shape, colour and luminance, might contribute to its saliency^12^. For example, Chamizo *et al*.^13^ demonstrated poor water maze performance using a small cue located further away from a goal but good learning with a near, big landmark. Indeed, cues that are located close to the goal gain more control over an animal’s performance compared to cues located further away^14,15^ (although see^16^). A recent operant model of spatial learning^2^, based on the general associative model of Rescorla and Wagner^17^, allows participants to learn about multiple cues when they encounter success or failure at a given location. Further, the cues, including the geometric shape of the testing arena, are all allowed to compete with other. However, this model does not speak to the role of distal cues *per se*, their features (brightness, size, colour, shape) or, importantly, the vector information (distance and direction) they provide^18^. During learning, some features, including independent distance and directional components, may be used while others not. Indeed, evidence for separate distance and directional components has been observed with bees^19^, pigeons^20^, Clark’s nutcrackers^21–23^ and rats^9,16^. Here we present an associative model of landmark learning, based on Rescorla & Wagner^17^, and combine this with an autoregressive random walk model of goal-directed swimming behavior^24^. With this novel integrated model we can separate distance and directional components of each cue, vary the relative salience and compare the location of each cue to examine how each component can affect spatial learning. By comparing the results of the model to experimental data, predictions as to the most effective strategy and how the task has been learnt can be revealed.

## Methods

### The water maze task

The water maze consisted of a black circular fibreglass pool (1.7 m diameter; 38 cm deep). Male Wistar rats (Charles River, UK), aged 3 months (250–350 g) were used as subjects (see^16^ for details). Animals escape the water by locating a platform (9 cm diameter, 29 cm height) located in the northeast quadrant of the pool. The platform was submerged 2 cm below the water, rendering it invisible when swimming. The pool was surrounded by a black curtain. Two light bulbs, one located in the northwest (NW, Far) and the other in the northeast (NE, Near) corner (relative to the platform), suspended on the inside of the curtains served as distal cues; we have shown that animals learn the task rapidly using these cue locations^9^. Animals were given 4 × 1 minute trials/day for 10 days to acquire the task. Animals were placed into the pool from the North, South, East and West positions in a pseudorandom order for each day. Escape time was used to assess learning. Retention was tested 24-hours post-acquisition where animals were allowed 1 minute to swim in a platformless pool. All animals for this trial were placed into the pool from the SW position (a novel starting position). Percentage time (of 60 seconds) in the target region (NE quadrant/area) was used to assess retention. All simulation experiments were matched to this experimental procedure, with respect to cue positioning, starting positions, number of trials, etc. All experiments were approved by Maynooth University Ethics Committee (BSRESC-2011-0015). Guidelines for the maintenance and experimentation of animals conformed to the Department of Health and Children (Ireland) guidelines under statutory instrument (S.I.) No. 543 of 2012 and the European directive 2010/63/EU.

### Modelling swimming behaviour in the water maze

**Figure 1:**
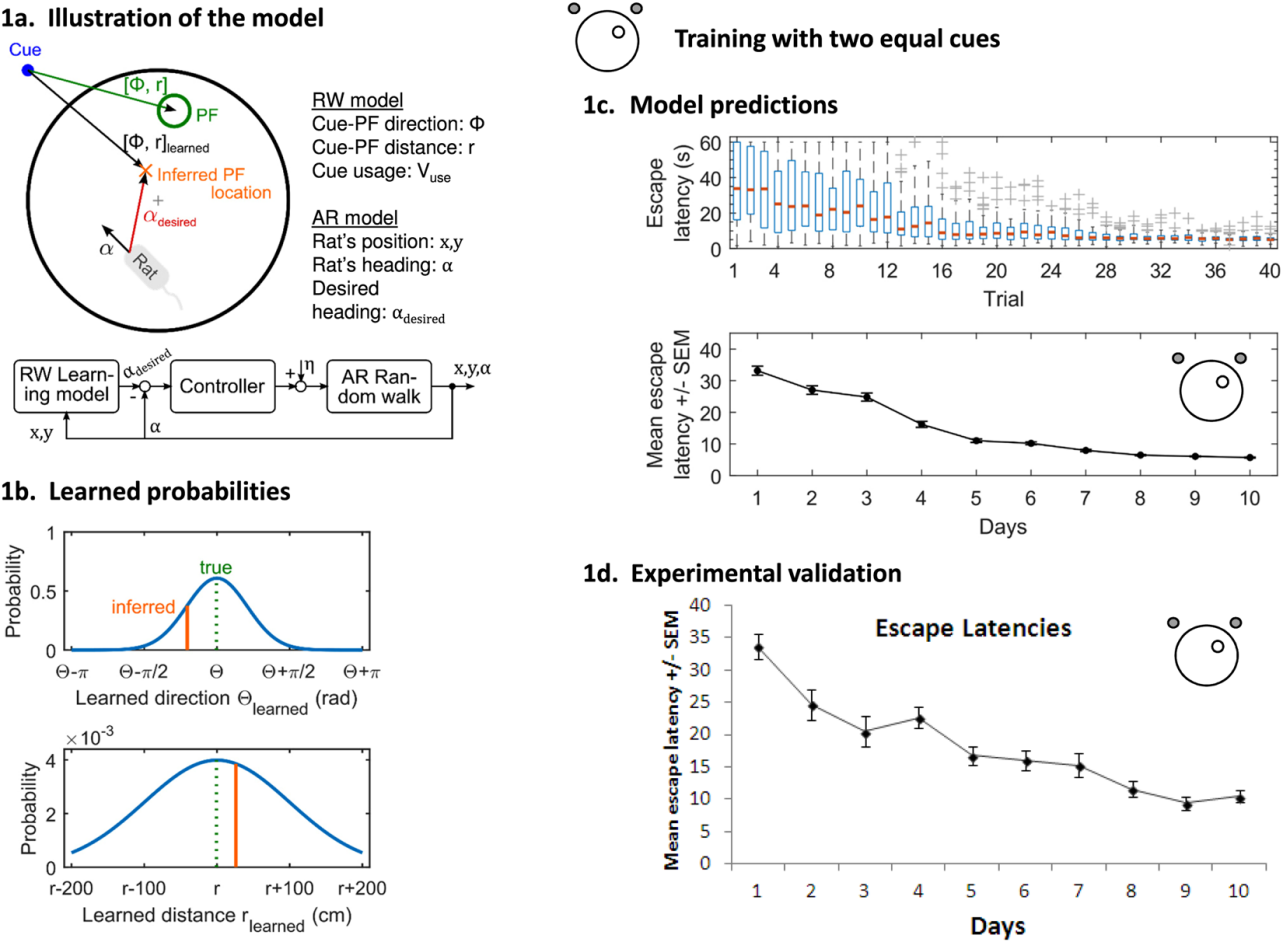
(**a**) The model consist of three components: (1) Autoregressive (AR) random walk describing the rats heading (α) and position (x, y), see Eqs 1 and 2; (2) controller adjusting the rat’s heading in each step, see Eqs 3 and 4; and (3) Rescorla-Wagner (RW) learning model providing the rats desired heading as the input for the controller, see Eqs 5 and 6. PF: platform. **(b)** Probabilities learned in the RW model. For each cue, the rat learns the direction and distance from the cue to the platform, see Eqs 7 and 8. The learned distributions are centred around the correct direction and distance values (dashed line). The more is learned, the narrower the distribution. In each swimming episode the rat guesses/infers the platform location by drawing random values from these distributions (red bar). **(c)** Simulated escape latencies using two cues of equal brightness. Top figure, boxplots (indicating upper and lower quartiles (blue box), median (red) and outliers (+marks) outside 1.5x the interquartile range (whiskers)) depicting decreases in the median escape latency across 40 trials of learning; middle figure, line graph depicting the equivalent decrease in mean escape latencies across 10 days of learning (4 trials/day); inset: representation of the water maze with the two cues (grey circles) located in the NE and NW regions surrounding a circular pool of water with the hidden platform located in the NE quadrant of the pool (white circle). **(d)** Line graph showing a decrease in mean escape latencies (with standard error bars) across 10 days of training (4 trials/day) with cues of equal brightness using experimental data taken from Farina *et al*. (2015). Inset: representation of the pool and cue locations.

Before examining a learning mechanism, we will describe a model of how an animal swims in the water maze (see^24^, Fig. 1a). Prior to training rats exhibit random movements, however with learning, these movements become increasingly goal-oriented. Between two measuring points, a rat moves a certain distance (step size) in a certain direction (heading). This movement can be described by a directed random walk, whereby the step size and heading change are random. Goal-oriented movements are enabled through a feedback control loop taking the rat’s desired heading as input. Briefly, the model consists of a directed random walk, in which the step size is a Rayleigh distributed random variable. The heading change is determined by an autoregressive (AR) model coupled to a control loop. The loop takes the rat’s desired heading as an input and consists of a linear filter providing an estimate of the control error and a proportional feedback gain. Mathematically, the random walk is given by

1 $$\begin{array}{rcl}{{x}}_{{t}} & = & {x}_{t-\Delta t}+{\Delta }{{r}}_{{t}}\,\sin \,{\alpha }_{{t}},\\ {y}_{t} & = & {y}_{t-\Delta t}+\Delta {r}_{t}\,\cos \,{\alpha }_{t},\\ {\alpha }_{t} & = & {\alpha }_{t-\Delta t}+\Delta {\alpha }_{t},\end{array}$$

where *x*_*t*_*, y*_*t*_ and *α*_*t*_ denote position and heading of the rat at time *t*. Δ*t* denotes the sampling time, i.e. the time required to perform one step. Δ*r*_*t*_ denotes the step size, which is a Rayleigh distributed random variable. The heading Δ*α*_*t*_ is given by the autoregressive model

2 $${\Delta }{\alpha }_{t}={{A}}_{1}{\Delta }{\alpha }_{t-\Delta t}+{A}_{2}{\Delta }{\alpha }_{t-2\Delta t}+{u}_{t},$$

where *A*_*i*_ are coefficients describing the relative contributions of the past values on the current value and *u*_*t*_ is an input term realizing the feedback control mechanism as follows

3 $${u}_{t}=K{\hat{e}}_{t}+{\nu }_{t}.$$

Here, *K* is a proportional feedback gain, ${\nu }_{t}$ a normal distributed random number and ${\hat{e}}_{t}$ the rat’s estimate of its heading error given by the low pass filter

4 $${\hat{e}}_{t}=(1-F){\hat{e}}_{t-\Delta t}+F\,({\alpha }_{desired}-{\alpha }_{t}),$$

where $0\le F\le 1$ is a weighting factor. See Supplementary Table S1 for parameter values and^16^ for details. We chose this model because it is a simple representation of the rat’s swimming behaviour in terms of mathematical complexity and it is well calibrated. All processes, distributions and parameters were identified from experimental data across conditions and simulations mimicked the experimentally-recoded swimming paths^9,24^.

### Modelling landmark learning in the water maze

We will base our learning model on the general principles of Rescorla and Wagner (RW)^17^. Formally, the model describes the amount of the expectation or associative strength V of the conditioned stimulus and how it changes in each trial. After each trial, the associative strength will have its old value plus the acquired change of value

$${{\rm{V}}}_{{\rm{new}}}={{\rm{V}}}_{{\rm{old}}}+{{\rm{\Delta }}{\rm{V}}}_{{\rm{i}}}.$$

The basic RW formula describes the change of V in each trial

$${\rm{\Delta }}{V}_{i}={\alpha }_{i}{\beta }_{j}({\lambda }_{j}-{V}_{{\rm{\Sigma }}}),$$

where Δ*V*_*i*_ is the change of associative strength of a conditioned stimulus i, *α*_*i*_ the salience of the conditioned stimulus, β_j_ the rate parameter of learning the unconditioned stimulus j, *λ*_j_ the maximum conditioning possible for the unconditioned stimulus and V_Σ_ the total associative strength. We use the above formalism to set up our model of learning. In this model the cues represent the conditioned stimuli, and learning to use the cues and their direction and distance information represent the unconditioned stimuli.

Learning to use the cues follows the RW model; one important aspect is that if there are several conditioned stimuli present, they compete for the animal’s attention, and learning will be based on the predictive sum of all stimuli^17^. Presenting two cues in the water maze resembles this situation and we have

5 $$\begin{array}{rcl}\Delta {V}_{1,use} & = & {\alpha }_{1}{\beta }_{use}({\lambda }_{use}-{V}_{\Sigma }),\\ {\rm{\Delta }}{V}_{2,use} & = & {\alpha }_{2}{\beta }_{use}({\lambda }_{use}-{V}_{{\rm{\Sigma }}}),\end{array}$$

where α_i_ is the salience of cue ${\rm{i}}\in \{1,\,2\}$, β_*use*_ the rate parameter of learning to use the cues, λ_use_ the maximum learning possible and ${V}_{{\rm{\Sigma }}}=\sum _{i}\,{V}_{i,use}$ is the sum of the associative strengths over all cues. Using the sum over all cues V_Σ_ means that the total available amount of associative strength λ_use_ is divided between the cues according to their saliences. Consequently, the change of the associative strength of a cue is not only dependent on its own value, but also the associative strength of the other cue. Therefore, if cue 1 was learned to 70% (V_1,use_ = 0.7) then the maximum possible learning value of cue 2 is 30% (V_2,use_ = 0.3).

Cues are critical in order to provide information as to where to search. If a cue lies at the goal, the animal simply has to head towards it. Most times, the goal lies at some distance and direction from the cues. Therefore, heading towards the cues is not sufficient^9^. The animal must incorporate this directional and distance (vector) information and move accordingly. Any model of landmark learning should not only consider how different cues may compete with each other, but also the vector information provided. We also use RW equations to model learning the direction and distance from each cue to the goal. Rather than describing the associative strength of the cues, we use the equations to describe the relative certainty of knowing the direction and distance. We assume that, in contrast to the associative strength of the cue-usage described above, the direction and distance information does not need to be distributed between the cues, but can be learned independently for each cue. The equations are

6 $$\begin{array}{rcl}\Delta {V}_{1,dir} & = & {\alpha }_{1}{\beta }_{dir}({\lambda }_{dir}-{V}_{1,dir}),\\ {\rm{\Delta }}{V}_{2,dir} & = & {\alpha }_{2}{\beta }_{dir}({\lambda }_{dir}-{V}_{2,dir}),\\ {\rm{\Delta }}{V}_{1,dist} & = & {\alpha }_{1}{\beta }_{dist}({\lambda }_{dist}-{V}_{1,dist}),\\ {\rm{\Delta }}{V}_{2,dist} & = & {\alpha }_{2}{\beta }_{dist}({\lambda }_{dist}-{V}_{2,dist}),\end{array}$$

where ${{\rm{\alpha }}}_{{\rm{i}}}\in \{1,\,2\}$ are the cue salience as above, β_dir_ and β_dist_ are the rate parameters for learning direction and distance information, and λ_dir_ and λ_dist_ are the maximal capacities to learn direction and distance, respectively. Note, learning the information of one cue does not impact on learning the information of the other cue, because unlike learning cue-usage, learning cue-information is not competitive. If the direction of one cue was learned to 100%, then the direction of the other cue can also be learned to 100%. All parameters of the model are provided in Table S1.

### Integrating the learning model with the swimming model

#### Inferring the platform location based on the learned direction and distance information from a cue

For each cue, the rat can guess the platform’s location, whereby the inferred platform location contains a random error that is determined by the learned association strengths. Formally, the associated uncertainties *σ*_*dist*_ and *σ*_*dir*_ are given by

7 $$\begin{array}{rcl}{\sigma }_{dist} & = & 1-{V}_{i,dist},\\ {\sigma }_{dir} & = & 1-{V}_{i,dir},\end{array}$$

where the index *i* denotes the selected cue. Based on these uncertainty values we can draw random numbers

8 $$\begin{array}{lll}{\epsilon }_{dist} &  \sim  & N(0,\,\,200\,{\sigma }_{dist}),\\ {\epsilon }_{dir} &  \sim  & N(0,\,75\,{\sigma }_{dir}),\end{array}$$

where *N*(0, *σ*) denotes the normal distribution with zero mean and *σ*^2^ variance. These random numbers provide the error for the inferred platform location, which is then given by

9 $$\overrightarrow{p}={\overrightarrow{p}}_{cue}+{\overrightarrow{x}}_{learned},$$

where the vector ${\overrightarrow{p}}_{cue}$ denotes the cue location and the vector ${\overrightarrow{x}}_{learned}$ given in polar coordinates

10 $${\overrightarrow{x}}_{learned}=(r+{\epsilon }_{r},\,\,\varphi +{\epsilon }_{\varphi })$$

describes the learned distance and direction from the cue to the inferred platform location.

#### Integrating the learning and swimming models

Combining the above described learning and swimming models yields an integrated model. In this model, the rat uses the learned direction and distance and information of the cues to infer the platform’s location. The direction to the inferred location is then used as the control input of the swimming model (Fig. 1a,b). Formally, this strategy is described by the following algorithm:

1. Pick a random cue and swim towards it. The probability to pick cue 1 is given by *V*_1,*use*_ and the probability to pick cue 2 is given by *V*_2,*use*_. Note that this choice is mutually exclusive and that the probability of picking both cues simultaneously is zero. Thus, the probability that no cue is selected is $1-{V}_{1,use}-{V}_{2,use}$.
2. If no cue is selected, then ${{\rm{\alpha }}}_{{\rm{desired}}}=0$ and the rat swims randomly for a certain amount of steps. After ${n}_{steps}=7$ steps, the random episode is terminated: start again with point (1).
3. If a cue was selected, then ${\alpha }_{desired}$ is given by the direction to the cue and the rat swims towards the cue for a certain amount of steps. After ${n}_{steps}=7$ steps, the cue-approach episode is terminated: continue with step (4).
4. Swim to the inferred platform location $\overrightarrow{p}$.
5. Start again with (1).

## Results

### Dissecting the model to predict how landmark learning is achieved

Using this model, we can dissect different elements of learning, analyse what information is learnt, and how effective is this information. Initially, we examined whether our model could simulate experimental spatial learning. Rats were simulated in a water maze using 4 trials/day for 10 days. Two distal cues of equal luminosity, located outside the pool in NW (Far) and NE (Near) locations, were used. The target goal was located in the NE quadrant. Figure S1 shows representative examples of simulated and experimentally recorded swimming paths in untrained and trained conditions. Figure 1c demonstrates how the simulated escape latencies evolved over training; Fig. 1d shows comparative decreases in escape latency from our experimental data^16^. As both simulated and experimental swimming paths and escape latencies showed good agreement, this gave us confidence in our model.

#### Acquisition

**Figure 2:**
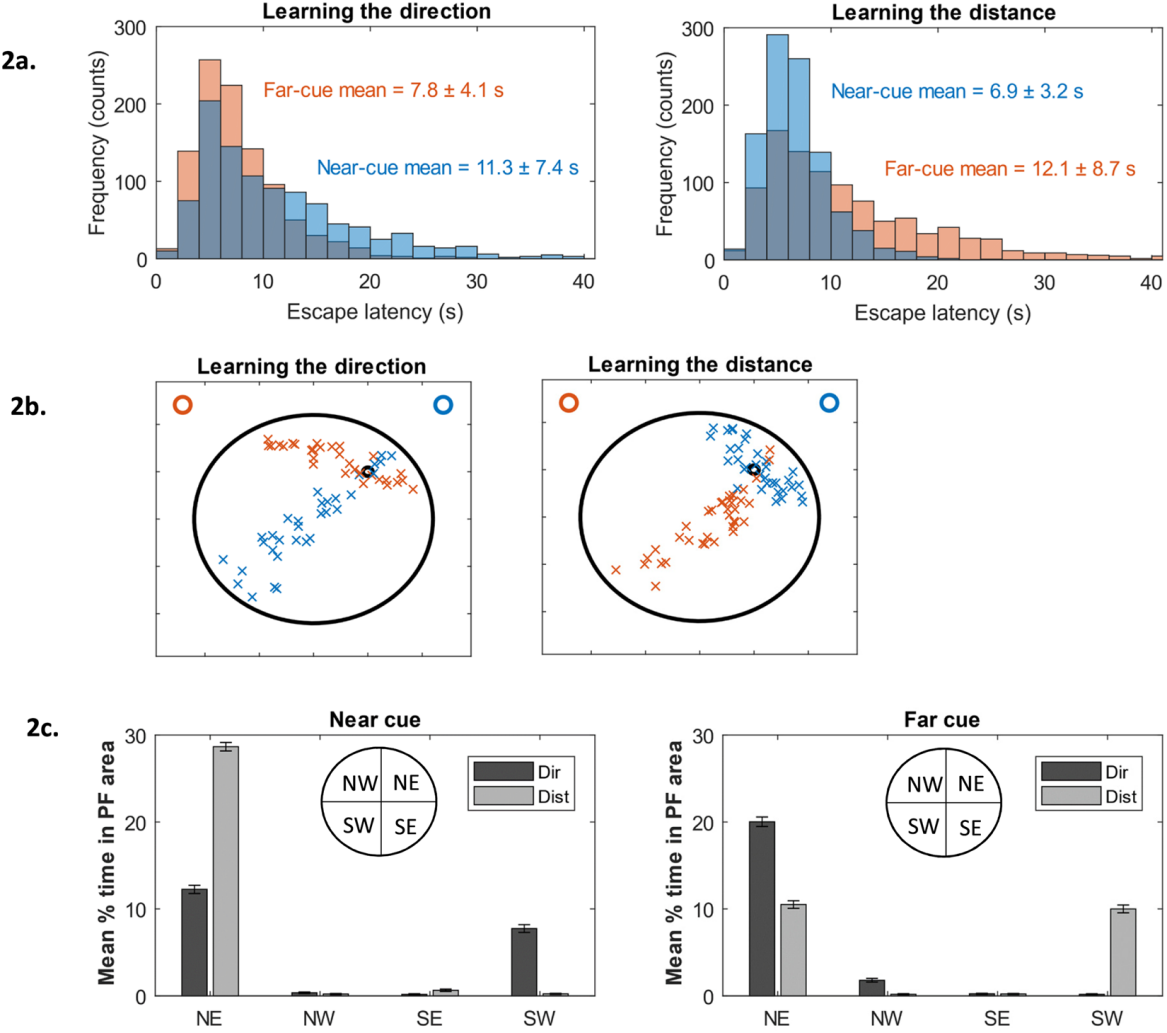
(**a**) Learning the direction (left panel) from the Far cue (red bars) results in shorter escape latencies compared to learning the direction from the Near cue (blue bars). Learning the distance (right panel) from the Far cue (red bars) results in longer escape latencies compared to learning the distance from the Near cue (blue bars). **(b)** Platform guesses using directional information (left panel) from the Far cue results in searching along the NW-NE axis (red crosses). Platform guesses using directional information from the Near cue results in searching along the NE-SW axis of the pool (blue crosses). Platform guesses using distance information (right panel) from the Far cue results in searching along the NE-SW axis of the pool (red crosses), while using distance from the Near cue results in searching confined mainly to the NE quadrant (blue crosses). Location of the two cues (Far and Near) represented by the red and blue circles, respectively. **(c)** Quantification of the platform guesses from (**b**) demonstrating differences in mean time spent in the NE target region depending on the location of the cue (Near vs Far) and whether direction (dark bars) or distance (light bars) information is used.

Equations 5 and 6 allow us to look at each cue separately and their directional and distance components. We initially examined how learning the *direction* from either cue would impact the escape latencies. We simulated 1000 rats to get an accurate estimate of how the escape latencies are distributed across trials. Learning the direction of the Near cue (NE) gives a relatively broad distribution with a mean escape latency of 11 s ± 7.7 s; whereas, learning the direction of the Far cue (NW) gives a much narrower distribution with a mean latency of 7.8 s ± 4 s (Fig. 2a, left panel). Thus, learning the direction of the Far cue is both more effective and more reliable than learning the direction of the Near cue. We then examined how learning the *distance* from either cue would impact escape latencies. Learning the distance from the Near cue gives a narrow distribution with a mean escape latency of 6.9 ± 3.25 s, but learning the distance from the Far cue gives a broader distribution (mean escape latency of 12.1 ± 8.3 s, Fig. 2a, right panel). These findings were further confirmed by examining the spatial distribution of platform-guesses rather than escape latencies. Platform guesses were obtained by drawing random numbers from the learned distance and directions distributions (Eqs 7–10) with an associative strength of ${{\rm{V}}}_{{\rm{i}},{\rm{dir}}}=0.95$ and ${{\rm{V}}}_{{\rm{i}},{\rm{dist}}}=0.95$ for learning the direction and distance, respectively. Figure 2b shows that learning either the direction of the Near cue (first panel blue crosses) or the distance of the Far cue (second panel red crosses) resulted in guesses along the south-west to north-east diagonal of the arena; learning either the direction of the Far cue (first panel red crosses) or distance of the Near cue (second panel blue crosses) resulted in platform guesses primarily in the target NE quadrant. The most accurate platform-guesses were obtained by learning the *direction* of the Far cue or learning the *distance* of the Near cue.

#### Retention

The above predictions are valuable, but individual trials vary greatly and large numbers of animals were need to get reliable estimates (e.g. 1000 rats were simulated for Fig. 2a). Such large numbers are not achievable with real animals. Therefore, we looked for less variable, more robust predictions that could be tested experimentally. Retention trials were simulated in which the platform was removed, the effectiveness of learning was assessed by measuring the time spent by animals in a circular area surrounding the platform location. We found that predictions of time-in-platform-area in retention trials were much less variable, with coefficients of variation (CV) about 10 times lower than for simulated escape latencies (CV = 0.32 for escape latencies, versus CV = 0.036 for time-in-platform-area). Using this method, we confirmed the aforementioned effects. Learning the distance of the Near cue (light bars, first panel) or the direction of the Far cue (dark bars, second panel) were the most effective strategies, resulting in animals spending more time searching in the NE quadrant (Fig. 2c). We note, however, that such results are dependent on the chosen cue location. For example, learning the distance from cues located in the eastern and southern positions result in searching in the NE/SE and NE/NW locations, respectively. Learning the direction from cues located in these positions result in searching in the NE and NE/SE locations, respectively (Fig. S2, left). Similarly, by keeping the Near cue in the NE position but shifting the Far cue to the SW position (rather than the NW above) also changes the searching dynamics depending on the use directional and/or distance information (Fig. S2, right). As the Near and Far positions that were used in the experimental set-up, we continued to use these locations for our subsequent simulations.

### Does changing the salience of the cues impact learning?

**Figure 3:**
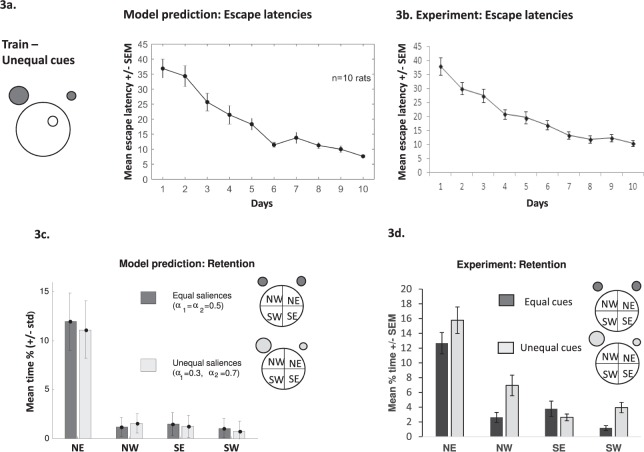
(**a**) Simulated escape latencies using two cues of unequal brightness. Inset: representation of the water maze with the two cues (grey circles) located in the NE and NW regions surrounding a circular pool of water with the hidden platform located in the NE quadrant of the pool (white circle). The larger grey circle represents the brighter cue. Line graph depicting the decrease in mean escape latencies of simulated animals across 10 days of learning (4 trials/day). **(b)** Line graph showing a decrease in mean escape latencies (with standard error bars) of experimental animals across 10 days of training (4 trials/day) with cues of unequal brightness. Simulated (**c**) and experimental (**d**) animals show good retention using cues of unequal (light grey bars) or equal brightness (dark grey bars) with both searching more in the targeted NE region compared to other equivalent areas of the pool. Experimental data is taken from Farina *et al*. (2015).

The model also allows us to attach different weightings to various cues. In a simple learning model the theoretical salience of a cue would be directly related to the cue’s strength in terms of physical properties (e.g. size, brightness, colour). In our model, α_i_ represents the salience of each cue (Eqs 5 and 6). Would changing the salience of the cues (e.g. setting α_1_ for the Near cue to 0.3 and α_2_ for the Far cue to 0.7) have an effect on simulated escape latencies and subsequent retention? How would the model results compare to experimental data? Our simulation (Fig. 3a) indicates that changing the saliency of the cues had little effect on escape latencies. This compares well to our experimental data (Fig. 3b), where the NW (Far) cue was brighter (40 Watt) than the NE (Near) cue (25 Watt). Therefore, all animals (simulated or experimentally) can learn the water maze task effectively with two equal cues (in terms of brightness) or if one cue is more salient than the other (examining Figs 3a,b and 1c,d). Similarly, altering the saliency does not affect retention; both model and experimental data show animals spending more time in the NE region compared to other areas (Fig. 3c,d).

**Figure 4:**
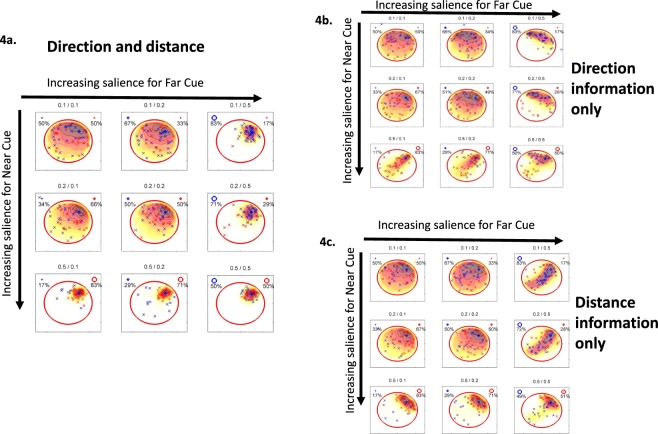
(**a**) Heat-maps showing the distribution of the inferred platform location (illustrative of how the pattern of searching changes) as the salience of one or both cues increases if both direction and distance is learned (**a**), just direction (**b**) or just distance (**c**). The percentage beside each cue refers to the relative saliency of one cue to the other, for example, if both are equal then the ratio is 50%:50%, if one is more salient than the other then the ratio is 83%:17%. The number above each plot refers to the strength of saliency with 0.1 being relatively weak which increases to 0.5 representing strong saliency. x-marks are examples of guessed/inferred platform locations based on the learned distance and direction information of the far (blue) and near (red) cue.

Given that knowing the distance or direction from a particular cue can affect an animal’s searching pattern, we then modelled where an animal might search if only *directional*, *distance* or *both* types of information was available from the cues, while simultaneously altering the salience weighting of each cue. Figure 4a shows that as the salience of both cues increases the searching pattern becomes more precise (along the diagonal from top left to bottom right). Irrespective of which cue is more salient, for example, the Far cue (top right panel; 83%:17%) or the Near cue (bottom left panel; 17%:83%), searching is very precise and is confined to the NE. Figure 4b shows how the searching pattern changes when only directional information is used, combined with salience change. As the Near cue becomes more salient searching spreads along the NE-SW axis (first column, top to bottom). As the Far cue becomes more salient searching is located mainly in the NW and NE regions (first row, left to right). Figure 4c shows the simulated pattern when only distance information is used, again combined with changing salience. As the Near cue becomes more salient searching is confined to the NE region (first column, top to bottom). As the Far cue becomes more salient searching is focused along the NE-SW axis (first row, left to right). These results suggest that enhancing the salience of either or both cues allows searching to become more precise. Although, enhancing the directional or distance information from a particular cue also increases accuracy (Fig. 4b,c), searching is more precise when both types of information are provided (Fig. 4a).

### Modelling what has been learnt

**Figure 5:**
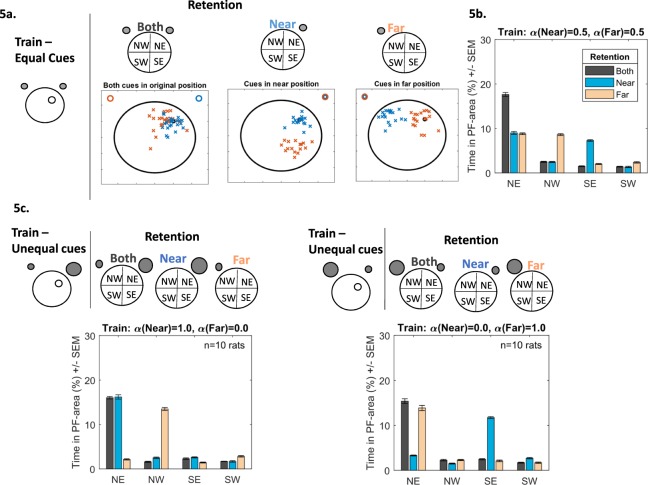
(**a**) Following training to find the hidden platform in the NE quadrant using two equally salient cues, retention is then tested in the presence of both cues (left), the Near (middle), cue only or the Far cue only (right). When the Near cue is presented, simulated animals alternate their searching between the NE and SE regions. In the presence of the Far cue searching is alternated between the NE and NW regions. In the presence of both cues animals search in the NE area only. **(b)** Bar chart depicting the search patterns during retention when both cues (black bars), the Near cue (blue bars) and the Far cue (light-red bars) are presented. **(c)** Changing the salience of the cue impacts the searching behaviour during retention. If the Near cue is more salient during training (left, top panel, represented by the larger grey circle), then presentation of the Near cue only during retention will result in searching in the NE quadrant (blue bars), presentation of the Far cue will be misinterpreted as the Near cue and will result in searching mainly in the NW (light-red bars). Alternatively, if the Far cue is more salient (right, top panel, represented by the larger grey circle), then presentation of the Near cue will be misinterpreted as the Far cue and will result in searching mainly in the SE (blue bars). Presentation of the Far cue will resulting in searching in the NE quadrant (light-red bars).
(ii) *Animals are able to distinguish individual cues and use the information pertaining each cue to solve the task* (*irrespective of whether one cue is more salient*). In this case the model predicts that animals would search in the target quadrant irrespective of which cue is presented (Fig. S3). Kamil & Jones^23^ showed this experimentally, whereby Clark’s nutcrackers were trained to find food located between a yellow and green pole, lying along the North-South axis. When tested with the green pole, birds searched north of the marker. When tested with the yellow marker, the birds searched south of the landmark; the birds searched at the correct location, irrespective of the landmark’s colour. Similarly, McGregor *et al*.^26^ showed in a water maze task that animals can readily find a hidden platform based on a single cue despite being trained with two cues. The authors further found that animals can use both local and distal cues with a minimum amount of interference between them.
(iii) *Animals learn information from the most salient cue and apply this knowledge, irrespective of what cue is presented*. The model can readily adjust the salience of one cue relative to the other by allowing a cue to have a maximum salience value of alpha = 1.0 and a minimum value of alpha = 0.0. Note, the value of one cue is dependent on the other, if one cue is 80% than the other cue is 20%. Figure 5c shows simulated searching following the salience adjustment for both Near and Far cues. When the Near cue has maximum salience (Fig. 5c left panel, α(Near) = 1.0, α(Far) = 0.0), the model predicts that, during retention, when presented with the Near cue (blue bars) searching will be in the NE. With the Far cue (pink bars), searching will be in the NW. Therefore, irrespective of the presented cue, it will always be interpreted as the most salient one (the Near one in this case) and search relative to this. Likewise, when the Far cue has maximum salience (Fig. 5c right panel, α(Near) = 0.0, α(Far) = 1.0), in the presence of the Near cue, searching will in the SE area (blue bars). In the presence of the Far cue, searching will be in the NE (pink bars). Here the presented cue is always interpreted as the Far one (the most salient cue in this instance). The simulated animal has generalised information relating to the salient cue to other available cues. With both cues present during retention, searching will be confined to the NE (black bars) area. When the cues approach equal salience (50:50) searching in the presence of a single cue will be in two areas (described in scenario (i) and Fig. 5b). While stimulus generalisation has been well documented experimentally and theoretically^27,28^, scenario (iii) suggests that animals either have not learnt any information pertaining to the less salient cue and/or that they cannot discriminate between the cues.

Examination of escape latencies or time-in-platform-area alone does not speak to *how* the animal has acquired the task. One method used in experimental psychology to examine what has been learnt, with respect to the cues, is to remove one of the cues following training. For example, Collett *et al*.^25^ trained gerbils to find food located near two landmarks. During re-test, one landmark was removed and the authors examined where the gerbils would search. By applying this method of cue removal following training to our model a number of scenarios can be contemplated:

(i) *Two cues are indistinguishable from each other*. Animals are trained with two indistinguishable cues. If one cue is removed during re-test the animal is unable to distinguish which has been removed. Therefore, the simulated rat assigns one of the learned cue-platform relationships to the available cue randomly (with probabilities proportional to the cues’ associative strengths), swims there and repeats the process until the trial is terminated. Thus, the simulated rat will use both Far and Near cue information in successive swimming episodes, looking for the platform in two different quadrants. For example, tested with the Near cue only (Fig. 5a, middle panel), animals interpret this as either the near (blue crosses) or the far (red crosses) one - the model predicts a searching pattern in NE and SE (Fig. 5b, blue bars). When tested with the Far cue (Fig. 5a, right panel), the animal interprets this as the near (blue crosses) and far (red crosses) cue equally, searching in NW and NE (Fig. 5b, pink bars). In the presence of both cues animals search in the NE (Fig. 5a left panel and Fig. 5b black bars). This pattern of searching has been observed by Collett *et al*.^25^. Gerbils were trained to locate food between two identical landmarks. Upon testing, one of the landmarks was removed, consequently the subjects searched in two locations.

### Experimental testing of the modelled scenarios

**Figure 6:**
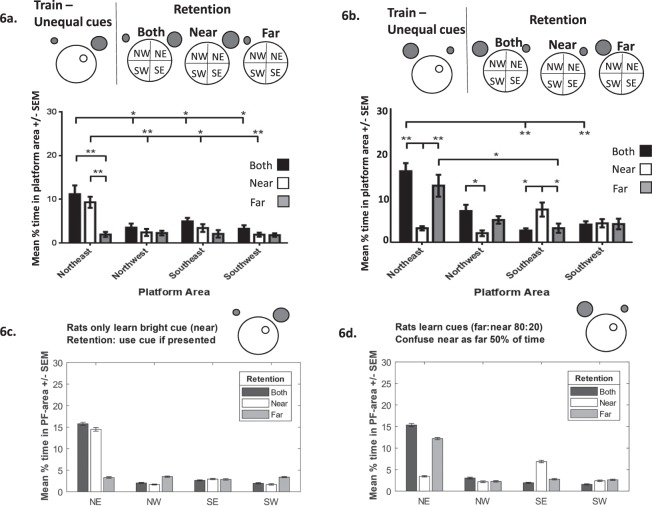
(**a**) Experimental data (from Farina *et al*., 2015) depicting the searching patterns of animals following training with two cues of unequal brightness (the brighter cue is located in Near position, represented by the larger grey circle). During retention when presented with the brighter Near cue animals search mainly in the NE quadrant (white bars). When presented with the dimmer Far cue animals’ search is random (grey bars). (**b**) Searching patterns of animals following training with two cues of unequal brightness (the brighter cue is now located in the Far position, represented by the larger grey circle). During retention when presented with the dimmer Near cue animals mainly search in the SE quadrant (white bars). When presented with the brighter Far cue animals search in the NE quadrant (grey bars). Figure adapted from Farina *et al*. (2015). (**c**) Simulation in which the rats ignore the dim-far cue (salience α_2_ = 0) explains the data in (**a**). Here the salience of the near-bright cue is so high (because of its location and brightness) that it completely dominates the dim-far cue. (**d**) Simulations in which the rats partially misinterpret the dim-near cue as the far-bright cue when they are only presented with the dim-near cue, explain the data in (**b**). In this model, the bright-far cue achieves salience because of its brightness (α_2_ = 0.8) but the near cue also achieves some salience because of its location (α_1_ = 0.2). As a consequence of its high salience, the bright-far cue is learned well, and when presented with the bright-far cue, the rats use it correctly (grey bars). In contrast, because of its low salience, the dim-near cue is not learned well, and the rat tends to misinterpret it as the far cue (50% of the time in this model, white bars).

In recent experiments^16^ we trained animals with 4 trials/day for 10 days with the hidden platform in the NE quadrant. Two cues were used, one located in the NW (Far) and the other in the NE (Near). In one experiment (Fig. 6a), the near cue was brighter (40 W) compared to the far one (25 W), in a second experiment the brighter cue was located in the far position (Fig. 6b, inset). Twenty-four hours post-acquisition animals were divided into control, Far and Near groups, and retested in a platformless pool. The time spent in the target (NE) and the other three equivalent areas (NW, SW, SE) were recorded. The control group was re-tested, as per acquisition, in the presence of the two cues. The Near group was re-tested with just the NE cue (bright in experiment 1 and dim in experiment 2). The Far group was re-tested with just the NW cue (dim cue in experiment 1 and bright in experiment 2). Figure 6a,b show the results of experiments 1 and 2, respectively. The control group in both experiments swam in the NE area. Irrespective of the location, the group retested with the brighter, more salient cue also searched in the target location, as predicted by the model (scenario (iii)). As also predicted with scenario (iii), there was a generalization effect when presented with the less salient cue (Fig. 6b, Near, white bars); animals searched in the SE. However, this effect was weak (compare Fig. 6b to Fig. 5c right and see also^29^). Further, the generalization effect was not consistent; it occurred in experiment 2 and not experiment 1, where we predicted animals (Far group) would search in NW area (compare Fig. 6a to Fig. 5c left). These results suggest an element of uncertainty, not contemplated by the modelled scenarios. One explanation is that *animals are able to discriminate between the cues but only information pertaining to the salient cue is actually learned* (with little or no information learned from the less salient cue - an interference by salience effect, see^11,16^). Therefore, when animals are presented with the less salient cue, they recognize it but do not know what to do. As such, they can either swim randomly around the pool (e.g. experiment 1, Fig. 6a) or use information from the most salient cue (to a degree) (Fig. 6b). Irrespective of the explanation, all introduce a level of uncertainty when the animals encounter the less salient cue, something not considered in the modelled scenarios.

Can the model accommodate this uncertainty? By relaxing the assumption that the cues cannot be distinguished, so that rats would, to some extent, know whether they are tested with a particular cue. If we set the simulation so that animals learn information from the bright cue (irrespective of its position). Then, the animal when presented with both cues (Fig. 6c black bars and S4a) or the bright one (Fig. 6c white bars – bright cue in Near position; Fig. S4a pink bars – bright cue in Far position) would search in the NE. When the dim cue is presented searching is random, irrespective of the location (Fig. 6c grey bars; Fig. S4a blue bars); they are confident when the bright cue is presented but uncertain about the dimmer cue. This uncertainty can be manipulated further by the model so that the animals may have ‘some’ knowledge about the dimmer cue (rather than nothing – especially if this cue is close to the goal). Therefore, if animals were trained with the bright cue in the Far position and the dim cue in the near position (Fig. 6d, inset), upon retention when presented with the dim cue only (Near group), the simulated animal could interpret this correctly as the dim cue 50% of the time but misinterpret it as the bright cue the other 50%. This reduces time searching in NE and increases time in the SE area (Fig. 6d, white bars). This generalisation effect critically depends on the relative salience of the bright cue. Decreasing the salience of the bright cue (in this case the far one), means that the other cue is used more and more time is spent in the NE area (Fig. S4b). This model provides a more nuanced explanation of the experimental data, accounting for uncertainty – a more realistic situation in biology.

### Modelling overshadowing directly

**Figure 7:**
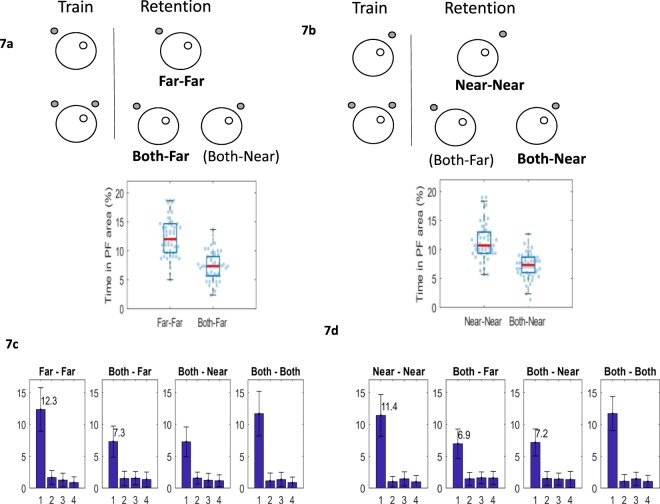
(**a**) Two groups of simulated animals were trained to find the platform in the NE quadrant. One group was trained and retested using the Far cue only (Far-Far), the second group was trained with both Near and Far cues and retested with each cue individually (Both-Far, Both Near). Bar chart depicting the percentage time spent by the Far-Far and Both-Far groups in the platform area during retention. Each dot is the simulated time-in-platform-area for a simulated rat (n = 50 simulated rats). Boxplots indicate median (red), upper and lower quartile (blue box). (**b**) Simulated animals were trained and re-tested using the Near cue only (Near-Near) or trained with both Near and Far cues and retested with each cue individually (Both-Far, Both Near). Bar chart comparing the percentage time spent by the Near-Near and Both-Near groups in the platform area during retention (n = 50 simulated rats). Boxplots indicate median (red), upper and lower quartile (blue box). (**c**) Mean % time in platform area (PF) for all quadrants (1 is the quadrant where the PF was located during training) for all retention groups (from **a** top): Far-Far, Both-Far, Both-Near, Both-Both. (**d**) Mean % time in PF for all quadrants for all retention groups (from **b** top): Near-Near, Both-Near, Both-Far, Both-Both. Error bars indicate standard deviation of the distribution (n = 50 simulated rats for each group).

Overshadowing is a key characteristic of associative learning^8,30^ and the above model and experimental data allows for this by adjusting the cue-usage, particularly seen above in scenario (iii). However, to show an overshadowing effect more directly, many experimental psychology experiments train two groups of animals on a particular task. The first group is trained using a compound stimulus (e.g. tone and light), while the second group is trained using just one stimulus (e.g. light). During retest both groups are presented with the light only; if animals in group 1 (tone and light) perform worse than group 2 (light only) it is suggested that overshadowing has occurred; therefore, it is suggested that the two stimuli in a compound gains less associative strength than each stimulus separately. Using a similar procedure, we asked whether we could simulate this experimental setup in the water maze task using our model. To do this, one group was trained to find the platform in the NE quadrant using just the Far cue (positioned as previously described) and the second group was trained with both the Near and the Far cues (see Fig. 7a, top panel). Figure 7a (bottom panel) shows that the group trained and re-tested with the Far cue (Far-Far) spent more time in the platform area than the group that was trained with both cues and re-tested with the Far cue only (Both-Far). The reverse was also found to hold true, whereby a group trained and re-tested with just the Near cue (Fig. 7b, Near-Near) spent more time in the platform region compared to the group that was trained with both cues and then re-tested with the Near cue (Both-Near). All contemplated scenarios comparing across all quadrants are shown in Fig. 7c,d. These findings suggest that our model provides a good account of overshadowing.

## Discussion

This paper present a novel and biologically realistic model of landmark learning accounting for many experimental findings across a range of species. The model modifies the classic Rescorla-Wagner learning model and integrates it with an autoregression random walk model. It is an associative model of learning whereby cues in the environment compete for associative strength. Unlike other models (e.g.^2^) that used a rectangular testing arena, where the shape can be used as an additional cue^31,32^, we have used the traditional circular water maze task. As such, it does not provide an obvious geometric cue. Despite this, we acknowledge that the side of the arena can provide some information that could help the animal to locate the goal (see^10,33,34^). In addition, Kealy *et al*.^35^ found that over-trained animals tended to swim around the side of the pool at a certain distance from its edge during a retention trial (in which the distal cues had been removed). This would suggest that during training, in the presence of distal cues, animals were able to learn something about the distance of the escape platform from the pool wall. Similarly, Shires & Aggleton^36^ were able to train animals to a platform located either 5 cm or 13 cm from the pool wall without the availability of extra maze cues. While not included here, the information provided by the pool wall can be easily incorporated into our model and allowed to compete with other cues.

A key component of our model is that each distal cue provides distance and directional information that can be examined independently, a concept supported by behavioural (in pigeons^20^; in honeybees^19^) and neural studies (e.g. head-direction cells^37^; grid and border cells^38^). In our setup, we show that directional information from a far cue leads to more accurate searching compared to the cue’s distance information. Whereas, distance information from the nearer cue proved to be better compared to its directional information. Although a number of studies have shown that near landmarks tend to gain strongest control over behaviour^5^, other studies, examining vector information directly, have shown that as a cue gets farther from the goal distance errors increase more rapidly than directional errors^21–23^. However, it is important to note that more recent findings by the same group^39^ suggest that this might not always be the case and in some circumstances, directional error may be larger than distance error as distances increase. Therefore, usefulness of a particular cue and whether one component gains greater control over the other may depend very much on the location of the cues relative to the platform (see also Fig. S2) and the experimental set-up. Future simulations should examine the effect of increasing the number of cues, as well as, varying the cues’ location, the size and shape of arena^31,32^. All these can be easily accommodated by our model, which in principle can be adapted to any species.

Location of cues is clearly a critical feature, but other components including the cue salience in terms of size, shape, brightness, colour and other features can also gain control over spatial behaviour. The model allows for this, whereby different cues can be weighted differently. So not only does a cue compete with other cues, as in the traditional associative mechanistic accounts of learning, but the information provided by the cues takes on a greater significance. Allowing a cue to have greater saliency results in animals using the information provided by that cue to a greater degree. Initial examination of escape latencies and retention searching patterns suggests that animals learn equally well irrespective of whether one cue is more salient than the other. In general, enhancing the cue’s saliency does enhance both the individual directional and distance components leading to more accurate searching; however, precise searching is found when the combined directional and distance information is available. Another important feature of the model is that it can be trained on various scenarios, so that what an animal has learnt can be dissected. For example, by removing one of the cues during retention, the search pattern of the modelled animal can be examined and compared to experimental findings. We were also able to adjust our model to account for uncertainty and subtleties found in biology, highlighting the flexibility of the model. Although we modelled a number of scenarios that provided a good account of water maze learning, generalised learning phenomena (e.g. stimulus generalisation^28,29^, overshadowing), as well as, data from different species (gerbils^25^; Clark nutcrackers^23^), many other scenarios can be contemplated. Furthermore, while the model tested scenarios that are based on an elemental account of learning, other accounts, such as configural learning^40^, whereby cues are associated with the goal in groups, could also be accommodated by the model. In summary, the model offers an associative account of spatial learning that adjusts the salience of individual cues and allows for the separation of cue directional and distance information, providing a fuller understanding of spatial learning and memory.

## Supplementary information

Supplementary information
