# Supplementary material for: Understanding the role of distance, direction and cue salience in an associative model of landmark learning
Source: Sci Rep. 2019 Feb 14;9:2026. doi: 10.1038/s41598-019-38525-7 (PMC6376129; doi:10.1038/s41598-019-38525-7)
Supplement: Supplementary file 1 — Supplementary information [file 41598_2019_38525_MOESM1_ESM.pdf]

Understanding the role of distance, direction and cue salience in an associative model of landmark learning.

Sean Commins[1*], Dirk Fey[2]

[1]Department of Psychology,
Maynooth University,
Maynooth,
Co. Kildare,
Ireland.

[2]Systems Biology Ireland
Conway Institute,
University College Dublin,
Dublin,
Ireland.

Contribution

SC: Formulated overall concept. Wrote and read paper.

DF: Designed the model, ran simulations. Wrote and read paper.

[*]Corresponding author; email: Sean.Commins@nuim.ie; fax: +353-1-7084767; telephone: +353-1-7084765.

Supplement 1. Example of a simulated rat's swimming path

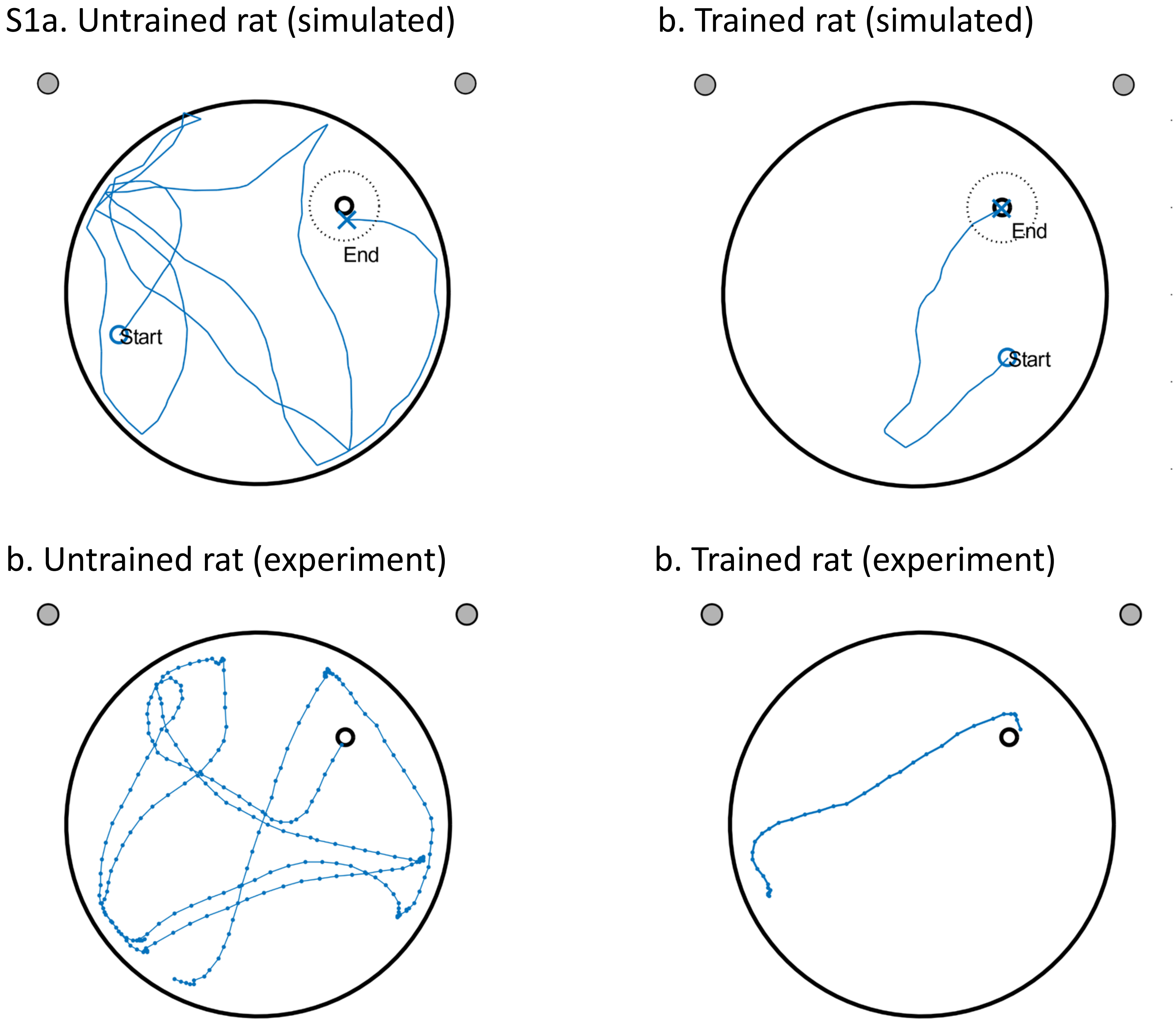

Supplement 2. Varying cue position changes searching dynamics depending on use of directional and distance information.

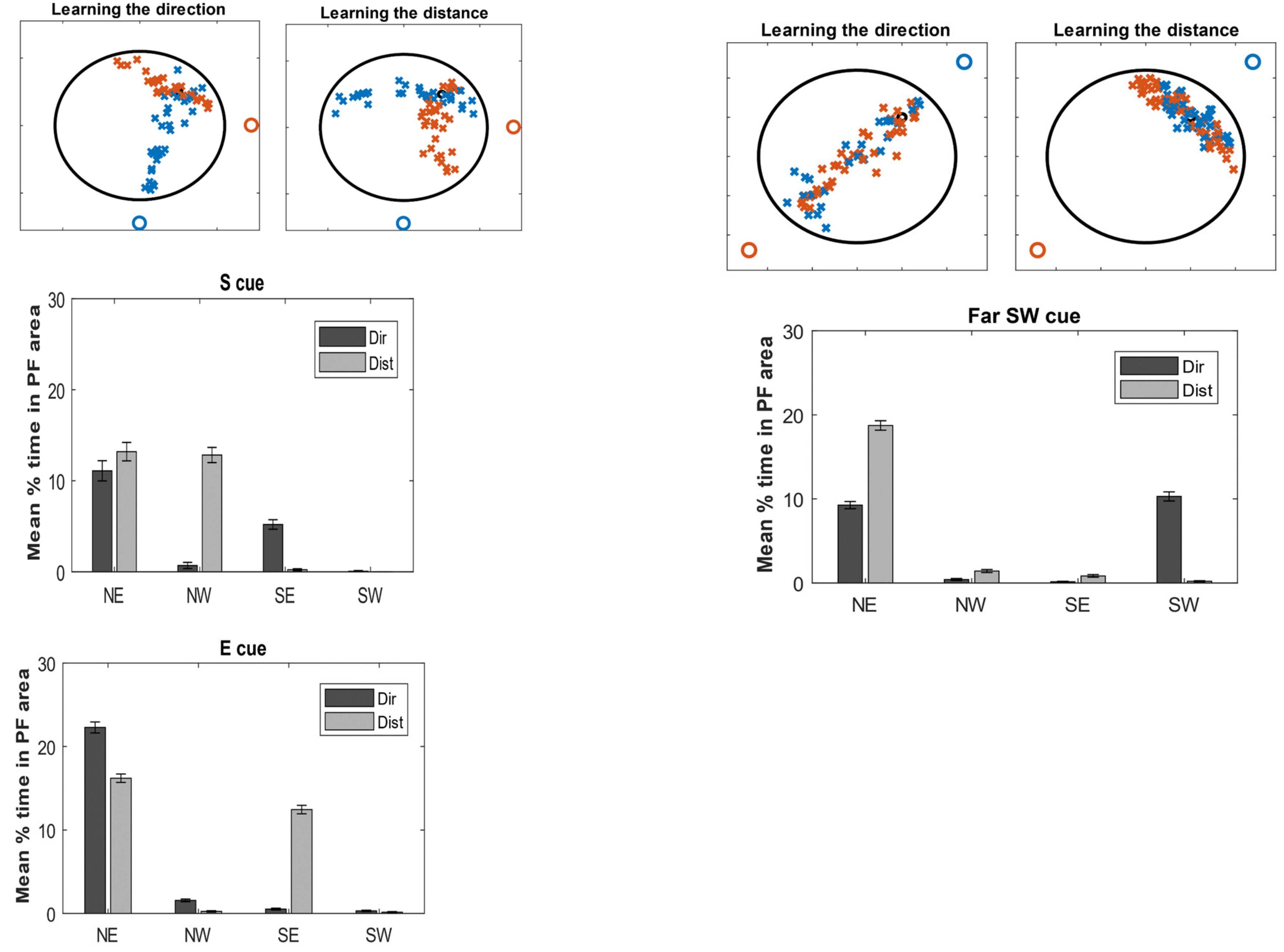

Supplement 3. Rat can distinguish cues independent of salience

S3. Equal cue saliences

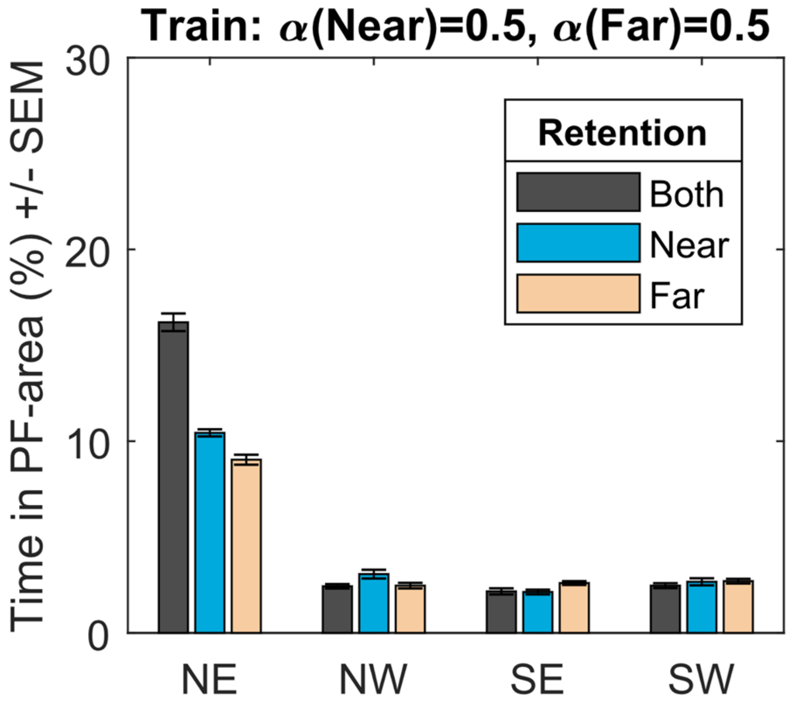

Supplement 4.

S4a.

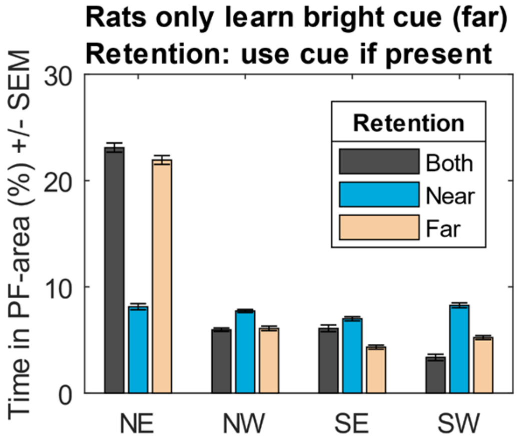

S4b.

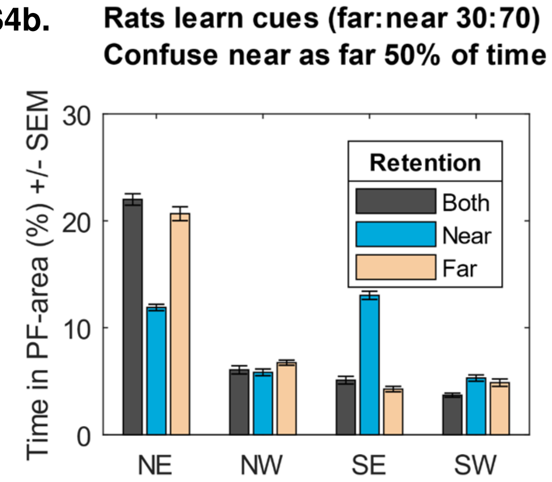

Supplementary Table 1

Swimming behavior model (AR random walk & controller)								
Parameter	A_1	A_2	F	K				
Value	0.174	0.153	0.95	0.5				
**Rescorla Wagner learning model**								
Parameter	α_1	α_2	β_{use}	β_{dir}	β_{dist}	λ_{use}	λ_{dir}	λ_{dist}
Value	0.5	0.5	0.2	0.2	0.2	0.33	1	1
